# High reliability and accuracy of dynamic magnetic resonance imaging in the diagnosis of cervical Spondylotic myelopathy: a multicenter study

**DOI:** 10.1186/s12891-022-06097-9

**Published:** 2022-12-20

**Authors:** Wook-Tae Park, Woo-Kie Min, Ji-Hoon Shin, Sang-Bong Ko, Eun-Seok Son, Jiyoun Kim, Jihoon Jang, Gun Woo Lee

**Affiliations:** 1 Department of Orthopedic Surgery, Yeungnam University College of Medicine, Yeungnam University Medical Center, 170 Hyonchung-ro, Namgu, Daegu, 42415 South Korea; 2 Department of Orthopedic Surgery, Kyungpook National Univeristy, Kyungpook National University Hospital, 130 Dongdeok-ro, Jung-gu, Daegu, 41944 South Korea; 3 Department of Orthopedic Surgery, Pohang Semyeng Christianity Hospital, 94-5 Daedo-dong, Nam-gu, Pohang-si, Gyeonsangbuk-do 37816 South Korea; 4 Department of Orthopedic Surgery, Daegu Catholic University, Daegu Catholic University Hospital, 33 Duryugongwon-ro 17-gil, Daemyeong-dong, Nam-gu, Daegu, 42472 South Korea; 5 Department of Orthopedic Surgery, Keimyung University, Dongsan Medical Center, 1035 Dalgubeol-daero, Sindang-dong, Dalseo-gu, Daegu, 42601 South Korea; 6 Department of Orthopedic Surgery, Kosin University, Gospel Hospital, 262 Gamcheon-ro, Seo-gu, Busan, 49267 South Korea

**Keywords:** Cervical spondylotic myelopathy (CSM), Cervical spine, Diagnosis, Magnetic resonance imaging (MRI), Dynamic, Reliability, Accuracy

## Abstract

**Background:**

Cervical spondylotic myelopathy (CSM) is a critical condition that results in significant neurologic deterioration. An accurate diagnosis is essential for determining its outcome and prognosis. The pathology is strongly associated with dynamic factors; therefore, dynamic magnetic resonance (MR) image could be crucial to accurately detect CSM. However, very few studies have evaluated the reliability and accuracy of dynamic MR in CSM. In this study, we aimed to compare intra- and interobserver reliabilities and accuracy of dynamic MR in detecting CSM using sagittal MR scans of the neck in the flexed, neutral, and extended position.

**Methods:**

Out of 131 patients who underwent surgical treatments for CSM, 107 were enrolled in this study. The patient underwent three-types of sagittal MR scans that were obtained separately in different neck positions (neutral, flexion, and extension postures). The MR scans of the cervical spine were evaluated independently by three spine professionals, on the basis of tabled questionnaires. For accuracy, we performed a receiver operator characteristic analysis, and the overall discriminating ability of each method was measured by calculating the area under the ROC curve. The Cohen’s kappa coefficient and the Fleiss-generalized kappa coefficient was used to the inter- and intra-observer reliabilities.

**Results:**

The intraobserver reliability (using the Cohen’s kappa coefficient) and interobserver reliability (using the Fless kappa coefficient) were respectively 0.64 and 0.52 for the neutral sagittal MR. The accuracy of neutral sagittal MR in detecting CSM was 0.735 (95% CI, 0.720 to 0.741) while that of extension sagittal MRI was 0.932 (96% CI, 0.921 to 0.948).

**Conclusions:**

Dynamic MR significantly showed better diagnostic reliability and accuracy in detecting CSM compared to conventional MR. In particular, extension MR scans could provide a more accurate diagnosis than other images.

## Background

Cervical spondylotic myelopathy (CSM) is a clinical manifestation of cervical spinal cord impairment related to the narrowing of the cervical canal, and it is due to compressive forces acting on the cervical cord [1–7]. Because the spinal cord is repeatedly injured during cervical motion, especially during extension, cervical cord compression is known to be aggravated in neck extension postures [1, 8–11]. Therefore, during the diagnosis and treatment of CSM, this unique feature of the cervical spine (i.e., segmental instability) should be considered.

Based on these peculiar characteristics, dynamic magnetic resonance (dMR) imaging was introduced to analyze the dynamic compression of the spinal cord [10, 12, 13]. Previous reports have shown that the extent of cord compression is better evaluated with dMR than with conventional MRI (cMR) [11, 14–19]. In addition, cMR scans are often supplemented with dMR scans when determining the surgical segments and selecting the surgical methods. dMR consists of flexion and extension sagittal MR scans of the cervical spine. A few previous studies have reported that among these dMR scans, extension sagittal MR (edMR) scans could be of value for the detection of CSM [14]. However, dMR is not a routine parameter considered during surgical decision-making for CSM. In addition, studies on the reliability of edMR in detecting CSM are still insufficient. Moreover, to date, no studies have been published on the diagnostic accuracy of each MR method in surgical decision-making for CSM.

In this study, 1) the reliabilities of cMR and edMR in surgical decision-making, and 2) the diagnostic accuracies of surgical decisions established using each MR method were compared using a multicenter survey. We hypothesized that dMR, especially extension sagittal MR in dMR, could guarantee a higher reliability and accuracy than cMR in the diagnosis and surgical planning of CSM.

## Methods

### Selection criteria

This study was approved by the institutional review board of our institution, and informed consent was obtained from all patients. A consecutive series of patients who underwent surgical treatment for CSM between January 2016 and December 2019 were enrolled in this study. The inclusion criteria were as follows: 1) confirmed CSM according to our diagnostic criteria described below, 2) underwent surgical treatment for CSM by the anterior or posterior approach, and 3) underwent cMR with dynamic flexion/extension MR (dMR) imaging before the operation. Exclusion criteria were as follows: 1) doubtful fusion status irrespective of the reason, 2) poor image quality or overlapping shoulders prohibiting measurement, and 3) fractures, infections, tumors, or deformities.

### Diagnostic criteria for CSM

**Fig. 1:**
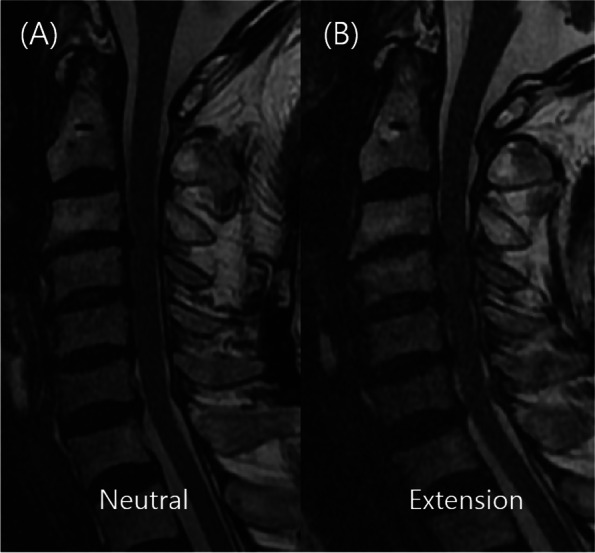
Case 19. Disagreement regarding the presence of CSM. **A** Neutral sagittal MR scan of the cervical spine. **B** Extension sagittal MR scan of the cervical spine. From the neutral image (**A**), all reviewers suggested that it was not CSM and that surgical treatment was unnecessary. However, from the extension image (**B**) they suggested that it could be CSM and that the patient needed to undergo a surgical treatment

**Fig. 2:**
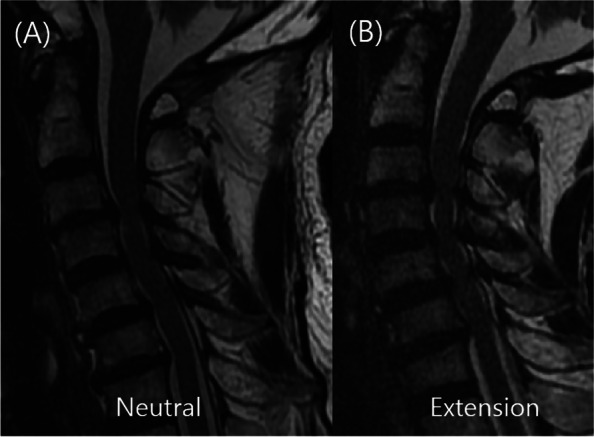
Case 41. Disagreement regarding the segments that needed a surgical treatment for CSM. **A** Neutral sagittal MR imaging of the cervical spine. **B** Extension sagittal MR of the cervical spine. From the neutral image (**A**), all reviewers suggested that the C3–4 segment is affected with CSM, and that it is where surgical treatment is indicated. However, from the extension image (**B**) they suggested that the C3–7 segments is rather involved and needs a surgical treatment

In our department, the CSM is diagnosed using a strictly guided diagnostic protocol. First, patients should have had specific symptoms when visiting the outpatient clinic. These symptoms included hand clumsiness, gait disturbance, hand or arm weakness, and other minor symptoms that have been described in the previous studies [1, 8, 9, 11]. In addition, they had positive signs of CSM on physical examination (including increased upper extremities reflexes, positive pathological reflexes, decreased performance on the grip and release test, and difficulties with tandem gait). If a patient was suspected to have CSM based on symptoms and/or physical examination, radiologic studies, such as cervical spine MR imaging, had to be performed to confirm diagnosis. In cervical MR imaging, sagittal MR scans are obtained separately in different neck positions (neutral, flexion, and extension postures) (Figs. 1 and 2).

To ensure that a fixed position was maintained during the procedures, we used a pillow (a size of 100x80x120mm) to flex the neck by placing underneath the most prominent area of the patient’s occiput or to extend the neck by placing underneath the most prominent spinous process of the cervical spine in the space between the bilateral trapezius muscles. This configuration of the pillow was strictly observed in all patients who underwent cervical spine MRI. When all the variables corresponded with the findings in CSM, the patient was diagnosed with CSM and managed accordingly. After surgery, the pre-operative diagnosis of CSM was re-evaluated using the Japanese Orthopedic Association (JOA) scores (comparison of the preoperative and 6-month postoperative scores) and central motor conduction test (CMCT) analysis. CMCT is a test based on motor evoked potentials (MEPs) and is used to determine the presence of pathologies in the brain or spine [12]. By evaluating the conduction velocity from the cerebral cortex to the spinal root, we were able to affirm whether or not a pathology was present between the brain and the spine. A delayed velocity can indicate the presence of a central nervous system disorder such as CSM.

### Study population

Out of the 131 patients who underwent surgical treatment for CSM during the reference period, 107 (46 men and 61 women) were enrolled in the study. The mean age was 58 ± 13 years (range, 29–84). The mean follow-up period was 29 months (12–53).

Anterior stabilization with a plate and screw system was performed in 39 patients, and posterior decompression with or without fixation was performed in the remaining 68 patients. Anterior cervical discectomy and fusion was performed in 34 patients and anterior cervical corpectomy and fusion in five patients. The posterior surgeries included cervical laminectomy without fusion in four patients, cervical laminectomy and fusion in 27 patients, and cervical laminoplasty in 37 patients.

### Radiologic measurement

The MR scans of the cervical spine were evaluated independently by three spine professionals, who were blinded to all the clinical information and basic characteristics of the patients. Tabled questionnaires were prepared for the three reviewers (Table 1). The questionnaires included 4 questions: (supposing the patient had typical symptoms and/or signs of CSM) 1) what is your diagnosis from the cervical spine MR?, 2) Is surgical treatment necessary for the pathology?, 3) What is your surgical approach for the patient (anterior or posterior)?, and 4) What is your surgical level for the patient? (Table 1).

**Table 1:** Questionnaires

	Question	Answer	Image 1 (neutral)	Image 2 (extension)
Case x (1st round or 2nd round)	1. What is your diagnosis from the cervical spine MR imaging?	CSM? Yes or No		
	2. Is surgical treatment necessary?	Yes or No		
	3. What is your surgical approach for the patient?	Anterior or posterior		
	4. What is your surgical level for the patient?	Levels		

Before performing the radiologic studies, they were taught the evaluation methods and were given a sample set of images, from 10 patients who were not included in this study, to evaluate. The most important images among the neutral and extension sagittal MRI scans were chosen by the corresponding author, and after ensuring that patients’ information were completely removed from the images, the images were stored in a single PowerPoint file that was sent to the three reviewers. The radiographs were reviewed in a random sequence, which was determined by a research coordinator who was blinded to the patients’ information. All three reviewers repeated the evaluations using the same radiologic images 2 months after the initial measurements to provide intraobserver data reliability while minimizing recall bias. During the second evaluation, information related to the previous results was not provided to the reviewers. All the evaluation data were independently tabulated by a blinded research coordinator.

### Statistical analysis

Statistical analyses were performed using MedCalc for Windows, version 12.3.0.0 (MedCalc Software, Mariakerke, Belgium). Statistical significance was set at a two-tailed *p*-value < 0.05. We performed a receiver operator characteristic (ROC) analysis for each MR view (neutral vs. extension) of the cervical spine for the diagnosis of CSM. The overall discriminating ability of each method was measured by calculating the area under the ROC curve (AUC) with 95% confidence intervals (CI) and statistically com-pared with the others. The discriminating performance of each method was assessed using the guidelines of Streiner and Cairney wherein an AUC between 0.50 and 0.70 was considered low; between 0.70 and 0.90, moderate; and over 0.90, high [20]. Regarding the questionnaire, the Cohen’s kappa coefficient was used to evaluate the intra-observer reliability of each cervical spine MRI view, and the Fleiss-generalized kappa coefficient was used to evaluate the interobserver reliability. The strengths of the inter- and intra-observer reliabilities were determined using the Landis and Koch criteria wherein a kappa value of 0.21 to 0.40 indicates fair reliability; 0.41 to 0.60, moderate reliability; 0.61 to 0.80, substantial reliability; and 0.81 to 1.0, excellent reliability [21].

## Results

### Reliability

Intraobserver reliability using the Cohen’s kappa coefficients was 0.64 for the neutral sagittal MR scans and 0.78 for the extension sagittal MR scans. Though these results indicated “moderate” reliabilities in the two groups, there was a statistically significant difference between the two groups (*p* = 0.03) (Table 2). Inter-observer reliability using the Fleiss kappa coefficient was 0.52 for the neutral sagittal MR scans (indicating “moderate” reliability) and 0.83 for the extension sagittal MRI scans (indicating “excellent” reliability) with a statistically significant difference between the two groups (*p* = 0.001) (Table 3).

**Table 2:** Cohen’s Kappa Coefficients for Intra-observer Reliability

Observers	Neutral MR image	Extension MR image
A	0.67 (0.53, 0.83)	0.84 (0.72, 0.95)
B	0.62 (0.46, 0.78)	0.77 (0.64, 0.90)
C	0.68 (0.54, 0.84)	0.78 (0.65, 0.90)
Mean	0.64	0.78

The numbers in parentheses indicate the 95% confidence interval

**Table 3:** Fleiss Kappa Coefficients for Inter-observer Reliability

	Neutral MR image	Extension MR image
First round	0.53 (0.43, 0.60)	0.86 (0.78, 0.88)
Second round	0.50 (0.47, 0.55)	0.81 (0.77, 0.89)
Mean	0.52	0.83

The numbers in parentheses indicate the 95% confidence interval

### Accuracy

For the accuracy in detecting CSM, the accuracy of neutral sagittal MR scans was 0.785 (95% CI, 0.760–0.813), which was “moderate,” and that of extension sagittal MR scans was 0.932 (96% CI, 0.921–0.948), which was “high.” There was a statistically significant difference between the two groups (*p* = 0.02) (Table 4). The sensitivity and specificity of each MRI modality for the radiologic evaluation of CSM are shown in Table 4. The sensitivity decreased while the specificity increased as the value increased for all the modalities. The sensitivities of neutral sagittal MR and extension sagittal MR were 81.4 and 93.8% respectively, and their specificities were 88.3 and 90.5%, respectively.

**Table 4:** Accuracy for the diagnosis of CSM

	Neutral MR image	Extension MR image
AUC^a^	0.785 (0.760, 0.813)	0.932 (0.921, 0.948)
Sensitivity (%)	81.4 (79.3, 87.6)	93.8 (88.5, 97.1)
Specificity (%)	88.3 (86.8, 92.0)	90.5 (87.7, 94.0)

The numbers in parentheses indicate the 95% confidence interval

^a^*AUC* area under the curve

## Discussion

The purpose of our multicenter survey was to compare the reliabilities of surgical decisions (regarding the surgical level and modality) set based on cMR and dMR and to compare the diagnostic accuracies of cMR and dMR by evaluating the improvement in clinical outcomes after surgical treatments indicated using preoperative plans set using each of the modalities. Our study showed that dMR resulted in more consistent inter- and intraobserver reliabilities, and more accurate diagnostic accuracy than cMR.

The pathophysiology of CSM includes both static and dynamic factor. Traditionally, the static factors have been the main issues considered in the development of CSM; these include congenital canal stenosis, osteophyte formation, facet joint hypertrophy, ossification of the posterior longitudinal ligament, ossification of the ligamentum flavum, and other minor static compressive lesions around the cervical spinal cord. For the static factors, some radiologic parameters have been developed, including the Pavlov ratio and sagittal canal diameter [22, 23]. However, to date, more focus is laid on dynamic factors which are now believed to also play a role in the development and aggravation of CSM. Dynamic compression of the cervical spine is highly related with buckling of the ligamentum flavum and segmental translational instability during neck motion, especially during neck extension. CSM from a dynamic compression can occur with dynamic cervical spine instability. It is defined as a translation of > 3.5 mm and an angulation of > 11° in flexion–extension views of a dynamic radiograph, that resulting in the narrowing of the spinal canal [3, 12, 14, 24]. Moreover, increased strain or shear forces of the spinal cord can induce axonal damage within the cord, increase membrane permeability, and cause conduction losses in myelinated axons. With repetitive compression or injury from the dynamic factors, irreversible loss of neuronal cells and ischemia could occur, thus contributing to the aggravation of CSM. Therefore, dynamic factors play crucial role in the induction and development of CSM; however, dynamic cord compression has rarely been defined clearly, except when there is confirmed instability of the cord segment on cervical spine radiographs.

Recently, dMR has been frequently used to evaluate static and dynamic spinal cord compression; though it is not yet a routinely requested work-up [14, 15, 25]. Clinical decision-making processes using edMR to evaluate the number of compression levels was introduced, and clinical decisions made based on edMR findings were reported to yield better results [26]. Some studies have reported the clinical applications of edMR in the determination of surgical levels, such as in cervical selective laminoplasty [16]. Regarding reliability, both intra- and interobserver reliabilities showed higher agreements concerning surgical segment choice and modality decision with dMR (especially edMR) than with cMR. From the outcomes of the intraobserver reliability, neutral sagittal MR showed moderate reliability, and extension sagittal MR showed moderate reliability. The two imaging modalities had statistically significant difference.; therefore, the authors suggested extension sagittal MRI could produce greater intraobserver reliability when compared to neutral sagittal MRI. Similarly, the results of intraobserver reliability also showed that extension sagittal MRI had a greater reliability than neutral sagittal MRI, and there was a statistically significant difference between the two imaging modalities (*p* = 0.001). Therefore, edMRI is expected to provide consistency in surgical planning for CSM and consequently, to reduce the variation of surgical outcomes among operators and institutions. In a previous study, Kim et al. confirmed that the diagnostic reliability of dMR was significantly higher than that of cMR [26]; however, their study only confirmed interobserver reliability and not intraobserver reliability. In addition, since the surgeons participating in this survey had completed a fellowship in spine surgery at the same institution, the rationale behind the surgical decision-making process could be the same. Because our survey was conducted on surgeons who had completed their education and fellowship in spine surgery at different institutions, the bias from uniform surgical decisions could also be minimized.

If CSM is not treated properly and in a timely manner, the patient’s symptoms gradually worsen, leading to negative consequences. Therefore, an accurate investigation of the structure and the level of the spinal cord compression should be established before the surgery. However, cMR does not reflect the movement of the cervical spine, such as segmental motion and instability, and can miss out some hidden levels of cord compression. The confirmation of diagnostic accuracy through the evaluation of the improvement of clinical outcome after surgery also showed that edMR has a significantly higher accuracy when compared to cMR. Specifically, neural sagittal MR provided “moderate” accuracy and extension sagittal MR provided “high” accuracy. More importantly, there was a significant difference between the two modalities (*p* = 0.02). This means that some segments (or patients) classified as being non-pathological on cMR developed dynamic cord compression, contributing to myelopathy symptoms, as being depicted at Figs. 1 and 2. In real situation, some patients showed typical symptoms and/or signs of CSM, but they had no typical CSM findings suggestive of CSM (such as significant cervical cord compression and snake-eye appearance of signal change). For such patients, establishing a proper surgical treatment plan in terms of surgical approach and surgical level is challenging. Thus, a surgical plan based on cMR is incomplete to treat spinal cord decompression. Consequently, there is a possibility that the patient’s postoperative outcome based on cMR be poorer than expected. In addition to increasing medical expenses, this can ultimately lead to socioeconomic problems such as delays in returning to previous jobs or unemployment due to reduced self-reliance. If dMR can detect “hidden cord compression lesions,” this will ensure high diagnostic accuracy, improve surgical outcomes, and reduce the socioeconomic burden. We confirmed that dMR has a positive effect on postsurgical outcome and prognosis. It also improves the diagnostic accuracy and helps in the selection of appropriate surgical treatment by revealing hidden cord compressions. Therefore, we believe that dMR, which reflects the daily movement of the cervical spine, should be performed prior to surgical planning.

This study has some limitations. First, the sample size was relatively small, making it prone to type 1 errors. Second, there was no clear flexion-extension position protocol for dMR. Third, because of the fundamental fact that MRI can only be done in the supine position, it was not possible to observe the actual cord compression in the erect position (under the effect of the weight of the head and gravity). Fourth, we did not include computed tomography (CT) scans of the cervical spine in the survey images of our study. Calcification of soft tissue structures around the spinal cord is an important factor in the decision on surgical method, and this is a clear limitation of our study. However, in order to include CT in our study design, the patient must be exposed to radiation twice before and after position change. As it is well known, it is very difficult to determine the degree of spinal cord compression from a CT image, so CT was not used because the benefit compared to the patient’s risk from radiation hazard was insignificant. Fifth, while conducting this study, we did not prepare for the fact that the patient’s CSM symptoms could worsen when the extension posture was applied. However, since edMR only acquires sagittal images for a short time after cMR imaging, no patients experienced worsening of symptoms during that time. Lastly, researchers should discuss the results and interpret them from the perspective of previous studies and working hypotheses. These findings and their implications should be discussed in the broadest context possible. Future research directions may also be highlighted.

## Conclusions

Dynamic MR showed a significant improvement in diagnostic reliability and accuracy of CSM compared to conventional MRI. In particular, extension MRI scans among dynamic MR scans might provide more accurate information to spine physicians regarding the establishment of the diagnosis, necessity of surgical treatment, approach method, and operative level of CSM than other images. Also, dynamic MR showed better results in diagnostic accuracy evaluated as a postoperative outcome. We therefore recommend the realization of routine dMRI, especially edMRI, before establishing the surgical plan for CSM.

## Data Availability

The datasets generated and/or analysed during the current study are not publicly available due [REASON WHY DATA ARE NOT PUBLIC] but are available from the corresponding author on reasonable request.
